# PREVALENCE OF AMBIVALENT AGEISM AMONG AGING SERVICES PROVIDERS AND ASSOCIATION WITH STRESS

**DOI:** 10.1093/geroni/igae098.3256

**Published:** 2024-12-31

**Authors:** Emily Kinkade, Heather Fuller

**Affiliations:** North Dakota State University, Fargo, North Dakota, United States; North Dakota State University, Fargo, North Dakota, United States

## Abstract

Ageism is an ambivalent construct consisting of positive and negative attitudes towards older adults. It is well documented that ageism is harmful to the physical and mental well-being of both the holder and target of ageist beliefs and behaviors. However, most ageism research focuses on hostile ageism within specific contexts (e.g., the workplace and medical settings). Little to no research investigates the prevalence and implications of ambivalent ageism among other populations that provide important resources to older adults. This study investigates how common ambivalent ageism is among aging service providers, and whether ambivalent ageist beliefs are associated with their feelings of stress and burnout. A small sample of Midwestern aging service providers (n=40) was recruited. Ambivalent ageism was measured using the Ambivalent Ageism Scale, while service provider stress and burnout were measured with the Perceived Occupational Stress Scale and the Copenhagen Burnout Inventory, respectively. A paired-samples t-test found that benevolent ageism was significantly higher than hostile ageism among aging service providers. Multiple regression analyses indicated that among aging services providers, benevolent and hostile ageism were associated only with personal burnout, and not with perceived stress, perceived occupational stress, nor work-related burnout. Such findings suggest that benevolent ageism may be more common among service providers because it is less likely to be viewed as problematic, and thus is more normalized and unquestioned. Further, ambivalent ageist beliefs may be associated with personal burnout due to exacerbating stress-related conditions such as compassion fatigue.

